# Comparison of Disseminated Histoplasmosis with and without Cutaneo-Mucous Lesions in Persons Living with HIV in French Guiana

**DOI:** 10.3390/jof6030133

**Published:** 2020-08-12

**Authors:** Sophie Morote, Mathieu Nacher, Romain Blaizot, Balthazar Ntab, Denis Blanchet, Kinan Drak Alsibai, Magalie Demar, Félix Djossou, Pierre Couppié, Antoine Adenis

**Affiliations:** 1 Service de Dermatologie-Vénéréologie, Centre Hospitalier de Cayenne, 97300 Cayenne, French Guiana, France; sophie.morote@ch-cayenne.fr (S.M.); romain.blaizot@ch-cayenne.fr (R.B.); pierre.couppie@ch-cayenne.fr (P.C.); 2 Centre d’Investigation Clinique Antilles Guyane, INSERM 1424, Centre Hospitalier de Cayenne, 97300 Cayenne, French Guiana, France; antoine.adenis@ch-cayenne.fr; 3 COREVIH Guyane, Centre Hospitalier de Cayenne, 97300 Cayenne, French Guiana, France; 4 DFR Santé, Université de Guyane, 97300 Cayenne, French Guiana, France; magalie.demar@ch-cayenne.fr (M.D.); felix.djossou@ch-cayenne.fr (F.D.); 5 UMR TBIP, Université de Guyane, 97300 Cayenne, French Guiana, France; denis.blanchet@ch-cayenne.fr; 6 Département d’Information Médicale, Centre Hospitalier de l’Ouest Guyanais, 97320 Saint Laurent du Maroni, French Guiana, France; b.ntab@ch-ouestguyane.fr; 7 Laboratory, Centre Hospitalier de Cayenne, 97300 Cayenne, French Guiana, France; 8 Service d’Anatomopathologie, Centre Hospitalier de Cayenne, 97300 Cayenne, French Guiana, France; mohamed.drakalsibai@ch-cayenne.fr; 9 Service des Maladies Infectieuses et Tropicales, Centre Hospitalier de Cayenne, 97300 Cayenne, French Guiana, France

**Keywords:** Disseminated histoplasmosis, HIV, Immunodeficiency, Cutaneous, Mucous, French Guiana

## Abstract

*Introduction*: Histoplasmosis is the main opportunistic infection and cause of death in HIV-infected persons living with HIV in French Guiana and probably in most of Latin America. The objective of the present study was to compare cutaneomucous histoplasmosis to non-cutaneomucous histoplasmosis in French Guiana. *Methods*: Between 1981 and 2014 AIDS-related disseminated histoplasmosis patients followed in the three hospitals of French Guiana were retrospectively studied. Only proven incident cases of histoplasmosis, either by pathology and/or mycological analysis, were considered. Mucocutaneous histoplasmosis was ascertained by a positive mucosal or cutaneous biopsy. *Results*: Thirty-one patients had mucocutaneous lesions, and 318 had no mucocutaneous lesions. Patients with cutaneomucous lesions were more likely to have had prior opportunistic infections (35.5%) than those who did not have cutaneomucous lesions (19.5%). They were more likely to be very severely immunocompromised (CD4 count < 50) (90.3% versus 62.8%) and less likely to have digestive signs (32.3% versus 74.1%) and superficial adenopathies (29% versus 50.2%) than those without cutaneomucous lesions. In terms of simple biological examinations, patients with cutaneomucous lesions had fewer signs of cholestasis. The diagnosis was significantly more likely to be performed by direct examination and pathology in those with cutaneomucous lesions than in those without such lesions. On the contrary, patients with cutaneomucous lesions were less likely to be diagnosed by fungal culture than those without cutaneomucous lesions. There was a greater but non-significant risk of early death in those with cutaneomucous lesions relative to those without (OR = 2.28 (95%CI = 0.83–5.7), *p* = 0.056. *Conclusions*: Mucocutaneous forms were associated with more profound immunosuppression and perhaps risk of early death. They are easily accessible for diagnosis.

## 1. Introduction

Histoplasmosis is the main opportunistic infection and cause of death in HIV-infected persons living with HIV in French Guiana [1,2]. This situation is presumably a reflection of what occurs in most of Latin America, where the disease is often undiagnosed and untreated largely because awareness and diagnostic tools are lacking [3]. The first differential diagnosis of histoplasmosis in French Guiana is tuberculosis [4]. The presence of mucocutaneous lesions is an important element to differentiate between these two diseases [5]. The cutaneous-mucous manifestations of histoplasmosis vary in their morphology and distribution. Papules, nodules, vesicles, erosive lesions, ulcers, or ulcerative cutaneous lesions, oral or nasal mucous ulcers have been described. Localized or diffuse lesions, multiple or isolated lesions can be observed [6,7,8]. Such polymorphism makes them non-specific clinical signs in AIDS patients [7]. However, they represent a very easily accessible site for minimally invasive biopsies, allowing a rapid diagnosis for a frequent cause of death among patients with advanced HIV [5]. Cutaneous-mucous forms of histoplasmosis are late presentations of the disease, appearing at the advanced stage of immunosuppression [7,9]. They are uncommon in the United States, but they are very frequently reported in Latin America [10,11,12,13,14,15,16,17,18]. This phenotypic difference between the two continents has raised the possibility of virulence differences between strains of histoplasmosis [7,15,19,20]. South American strains are considered more dermotropic than North American strains [20]. However, these geographic differences in cutaneous manifestations may have been confounded by access to diagnosis and care, and thus mostly reflected diagnostic delays [5,21,22]. In practice, for clinicians, apart from a rapid and simple diagnostic opportunity, the prognostic significance of cutaneous and mucous presentations is not clear and to our knowledge, few studies have compared disseminated histoplasmosis with and without cutaneomucous lesions. The main objective of this study was thus to compare cutaneomucous and non-cutaneomucous forms of HIV-associated histoplasmosis in French Guiana.

## 2. Materials and Methods

### 2.1. Study Design

The study was observational, retrospective, multicentric and spanned the period between 1 January 1981 and 1 October 2014. The patient data was entered prospectively but the data analysis was retrospective.

### 2.2. Study Population

The target population was co-infections with HIV and histoplasmosis included in the Histoplasmosis and HIV database of French Guiana. The source population was all known HIV patients followed in one of the three hospitals in French Guiana. The inclusion criteria were: Age > 18 years; Confirmed HIV infection; First proven episode of histoplasmosis either by direct mycological examination, culture mycological or histological examination (excluding PCR) performed on a variety of different samples (plasma, myelogram, digestive biopsies, skin biopsies, bronchoalveolar lavage, etc.) following EORTC/MSG criteria [23,24]. Patients with HIV and a suspicion of infection also benefited from thorough investigations searching for other fungi, parasites, bacteria, or viruses.

Recurrent histoplasmosis, unproven histoplasmosis (successful empirical antifungal therapy), or diagnosis based solely on the positivity of the PCR were not considered.

### 2.3. Judgment Criteria

The primary endpoint was defined by the presence versus the absence of *Histoplasma capsulatum*, on skin and/or nasal or oral mucosa by direct examination mycological and/or mycological culture and/or anatomopathological examination of tissues (EORTC/MSG criteria [23]). Histoplasma antigen detection assays were not available for diagnosis throughout the study period. The comparison groups were thus presence versus absence of cutaneous or mucous lesions in an HIV-infected person with disseminated histoplasmosis, which was defined as by the presence of the fungus in more than one location. Mortality within 30 days after initiation of antifungal treatment was the prognosis endpoint.

### 2.4. Study Conduct

The HIV-Histoplasmosis database was created in 1992. It included incident cases of Histoplasmosis in HIV-infected patients in the three hospital centers of French Guiana (Cayenne, Kourou, and Saint Laurent du Maroni). Epidemiological, clinical, paraclinical, immunovirological and therapeutic data were collected until 10-2014 on a standardized form and entered in the database. The data collected concerned all incident episodes of histoplasmosis in HIV-infected patients, previously known to be HIV positive or concomitantly discovered, hospitalized in one of the three above hospitals. The variables were the following: Socio-demographic data: sex, age, place of birth; Clinical data: symptoms on admission, clinical entrance examination; immunovirological assessment, standard biological examinations; medical imaging, mycology, pathology; treatment received, duration, dosage, route of administration; Survival data during the study period and 30 days after the start of antifungal treatment.

### 2.5. Statistical Analysis

The statistical analysis was performed with STATA^©^ software. (College Station, TX, USA) Frequencies and proportions were calculated for categorical variables. Means, medians, standard deviations, interquartile ranges, were calculated for quantitative variables depending on their distribution.

Standard hypothesis testing: Chi2 test or Fisher’s exact test for categorical variables, and the trend Chi2 for ordinal variables were used; for quantitative variables the Student’s *t*-test or the Ranksum test were used depending on their statistical distribution. The statistical significance level was *p* < 0.05.

### 2.6. Ethical and Regulatory Aspects

All HIV-infected patients were included in the DMI-2 database, administered by the Regional Coordination for the fight against HIV (COREVIH). This database is included in the French Hospital Database on HIV (FHDH) the national cohort of patients living with HIV. In French Guiana, socio-demographic, clinical and biological data, and therapeutics have been prospectively included since 1 January 1992. This database has received approval by the French regulatory authority, the National Commission Informatique et Libertés (CNIL) on 27 November 1991. All included patients are given oral and written information and they signed an informed consent. The main objective of the FHDH hospital cohort is to study the evolution, morbidity and mortality of patients living with HIV. The 1992 Histoplasmosis and HIV anonymized database was also approved by the CNIL (N° JZU0048856X, 07/16/2010), the French National Institute of Health and Medical Research institutional review board (CEEI INSERM) (IRB0000388, FWA00005831 18/05/2010), and by the Comité Consultatif pour le Traitement de l’Information pour la Recherche en Santé (CCTIRS) (N° 10.175bis, 10/06/2010).

## 3. Results

### 3.1. Cutaneomucous Lesions

Among 349 cases of disseminated histoplasmosis, there were 31 cases of cutaneous and/or mucosal histoplasmosis between 1 January 1981 and 1 October 2014. Of the 31 cases of cutaneo-mucous histoplasmosis observed during the study period, 15 were skin lesions, 13 were mucosal lesions and three simultaneously had cutaneous and mucous lesions. The most frequent skin lesions were papules (N = 14, 78%), followed by three ulcers and one nodule. Most lesions were diffuse and frequently localized on the face (14/18). The most frequent mucosal manifestations were ulcerative lesions (N = 10, 67%), sometimes both vegetative and ulcerative (N = 3, 20%), lesions were mostly oral (N = 10, 62%) often affecting the palate (7/16), and lips (N = 8, 50%). There was only one case of a nasal mucosal lesions. Two cases of palatal fistulas were reported.

Table 1 compares epidemiologic, clinical, biological, and therapeutic features of disseminated histoplasmosis with and without cutaneo-mucous lesions. Overall, most cutaneomucous forms were described in Cayenne, which is the dermatological reference center in French Guiana. Patients with cutaneomucous lesions were more likely to have had prior opportunistic infections than those who did not have cutaneomucous lesions. They were more likely to be very severely immunocompromised (CD4 count < 50) and less likely to have gastrointestinal signs and symptoms and superficial adenopathies. In terms of simple biological examinations, patients with cutaneomucous lesions had fewer signs of cholestasis (lower GGT and alkaline phosphatase concentrations). The diagnosis was significantly more likely to be performed by direct examination and pathology in those with cutaneomucous lesions than those without such lesions. Conversely patients with cutaneomucous lesions were less likely to be diagnosed by fungal culture than those without cutaneomucous lesions. There was a linear trend suggesting that those with cutaneomucous lesions had a smaller number of different sampling sites than those without cutaneomucous lesions (chi-square for linear trend *p* = 0.047) Overall, patients with cutaneomucous lesions were less likely to receive amphotericin B(liposomal or deoxycholate) than those without cutaneomucous lesions (OR = 0.4 (95%CI = 0.16–1), *p* = 0.04). Given the small number of cases, and some missing values multivariate analyses were not performed.

### 3.2. Case Fatality at One Month

There were eight early deaths out of the 31 cases of mucocutaneous histoplasmosis listed, thus a one-month fatality rate of 25.8% among these forms over the entire period studied. (Table 1) The proportion of early deaths (<1 month after antifungal treatment initiation) was greater in cutaneomucous forms but this failed to reach statistical significance (OR = 2.28 (95% CI = 0.83–5.7), *p* = 0.056. Given the small number of observations, multivariate analyses were not performed.

## 4. Discussion

The present study shows the differences between patients with disseminated histoplasmosis with and without cutaneomucous lesions. Patients with cutaneomucous lesions were generally more immunocompromised, more likely to have had a history of prior opportunistic infections, and less likely to have biological cholestasis than those who did not have cutaneomucous lesions. By contrast, they were less likely to have gastrointestinal signs and symptoms and superficial adenopathies. Unsurprisingly, given the accessible nature of such lesions and the absence of requirement of invasive sampling techniques, the diagnosis was more frequently performed by direct examination and pathology in those with cutaneomucous lesions than those without such lesions. Conversely patients with cutaneomucous lesions were less likely to be diagnosed by fungal culture than those without cutaneomucous lesions. The number of sampling sites tended to be lower in those with than in those without cutaneomucous lesions suggesting they received fewer explorations aiming to identify the pathogen. Patients with cutaneomucous lesions were less likely to receive liposomal (available in 2003) or deoxycholate amphotericin B than those without cutaneomucous lesions. The frequently subdued presentation may lead to the reluctance of clinicians to use potentially nephrotoxic drugs for what presents like a cutaneous problem. Finally, case fatality at one month seemed more frequent, this was however not statistically significant at the 5% cutoff point.

The study limitations are linked to the small number of cases of cutaneomucous lesions, the retrospective nature of the hospital cohort data collection and the clinically driven explorations that may have varied from case to case. Moreover, although there are many cases of histoplasmosis elsewhere [25], the majority of cutaneous and mucous lesions were diagnosed in Cayenne, which may reflect a bias due to the hospitalization of patient in the dermatology department in Cayenne. In addition, most cases were diagnosed in the 1990s because earlier HIV testing, HIV treatment, and fungal diagnostic progress allowed to avoid extensive dissemination after 1997. Our reasoning was cutaneous and mucous lesions were similar in the sense they had visible and accessible lesions. However, it is arguable that mucous and cutaneous presentations are different diseases and should not be pooled in the same group. Finally, given the small number of cases, and some missing values multivariate analyses were not possible to adjust for confounding factors. Despite, these weaknesses, to our knowledge this is the first study to compare disseminated cases with and without cutaneomucous lesions.

## 5. Conclusions

In conclusion, mucocutaneous forms of histoplasmosis were polymorphic, preferably on the face, trunk, and limbs. They should orient physicians toward the possibility of this diagnosis and are an easily accessible site to search for the fungus on direct examination of strained samples. They were less often associated with digestive signs or lymphadenopathies than non cutaneomucous forms. Compared with other forms histoplasmosis, they appeared as late-onset forms of HIV infection, in patients more profoundly immunocompromised, and perhaps at greater risk of death.

## Figures and Tables

**Table 1:** Comparison of disseminated histoplasmosis with and without cutaneomucous lesions.

Patient Features	Disseminated Histoplasmosis *with* Cutaneomucous Lesions N = 31	Disseminated Histoplasmosis *without* Cutaneomucous Lesions N = 318	*p*
**Sociodemographic**			
Mean age ((SD)) in years	41.1 (10)	40 (9.7)	0.53
Sex ratio (Male/Female)	2.9	1.8	0.26
Geographic origin, N (%)			0.05
French Guiana	10 (32.3)	89 (28.2)	
Haiti	10 (32.3)	71 (22.5)	
France	4 (12.9)	7 (2.2)	
Brazil	3 (9.7)	43 (13.6)	
Suriname	2 (6.4)	72 (22.8)	
Guyana	1 (3.2)	20 (6.3)	
Other	1 (3.2)	14 (4.4)	
Median number of years in French Guiana (IQR)	18 (7–32)	25 (10–38)	0.27
Inclusion center N (%)			<0.001
Cayenne	29 (93.6)	184 (57.9)	
Kourou	1 (3.2)	37 (11.6)	
Saint Laurent	1 (3.2)	97 (30.5)	
**HIV infection data**			
Transmission mode			<0.001
heterosexual	24 (77.4)	296 (93.1)	
Homosexual	3 (9.7)	4 (1.3)	
IV drug use	2 (6.5)	1 (0.3)	
Transfusion	1 (3.2)	0 (0)	
unknown	1 (3.2)	17 (5.3)	
HAART on admission	6 (19.3)	34 (10.7)	0.14
Primary prophylaxis on admission	2 (6.4)	40 (12.6)	0.56
Histoplasmosis is AIDS-classifying event	21 (67.7)	258 (81.1)	0.07
History of opportunistic infection	11 (35.5)	62 (19.5)	0.037
Concomitant opportunistic infection	9 (29)	128 (40.2)	0.22
Median CD4 count on admission (IQR)	30 (15–42)	32 (11–73)	0.27
Immunosuppression stage			0.01
CD4 ≥ 500 per mm^3^	0	2 (0.6)	
CD4 (200–500) per mm^3^	1 (3.2)	18 (5.8)	
CD4 (50–200) per mm^3^	2 (6.5)	96 (30.8)	
CD4 < 50 per mm^3^	28 (90.3)	196 (62.8)	
**Clinical and paraclinical examination on admission**			
Impaired WHO general performance score (>2)	12 (38.7)	131 (47.6)	0.34
Fever	26 (83.9)	284 (89.6)	0.36
Respiratory signs	18 (58.1)	148(46.7)	0.22
Dyspnea	9 (29)	48 (15.1)	
Cough	13 (41.9)	123 (38.8)	
Gastrointestinal signs and symptoms	10 (32.3)	235 (74.1)	<0.001
Abdominal pain	10 (32.3)	122 (38.5)	
Diarrhea	2 (6.4)	122 (38.5)	
Hepatomegaly	3 (9.7)	95 (30)	
Splenomegaly	3 (9.7)	53(16.7)	
Superficial adenopathies	9 (29)	159 (50.2)	0.02
>2 cm	4 (12.9)	61 (19.2)	
Radiological pulmonary involvement	16 (53.3)	147 (51.4)	0.84
Interstitial syndrome	16 (53.3)	118 (41.3)	
other	4 (13.3)	68 (23.8)	
Endoscopic digestive histoplasmosis			1
Upper digestive tract	1/7 (14.3)	13/75 (17.3)	
Lower digestive tract	2/3 (66.7)	56/86 (65.1)	
Standard biological tests on admission			
Hemoglobin (Laboratory normal values: 13.5–17.5 g/dL for men, 12.0–15.5 d/dL for women)			
Mean ((SD))	8 (2)	9 (1.9)	0.94
N (%) < 10 g/dL	17 (63.4)	219 (70.9)	0.55
Neutrophils (Laboratory normal values: 1500–7000/mm^3^)>			
Median ((IQR))	1998 (1600–3070)	2150 (1420–3500)	0.97
N (%) < 1000/mm3	2(7.7)	39 (12.7)	0.75
Platelets (Laboratory normal values: 150,000–400,000/mm^3^)			
Median ((IQR))	181,500 (81,000–235,000)	189,000 (108,500–273,150)	0.45
N (%) < 100,000/mm^3^	8 (30.8)	65 (21.1)	0.32
Serum creatinine (Laboratory normal values: 61.9–114.9 µmol/L for men and 53–97.2 µmol/L for women)			
Median ((IQR))	80 (79–106)	79 (65–96)	0.18
N (%) > 2N	2 (6.7)	15 (4.8)	0.65
Albumin (Laboratory normal values: 3.4–5.4 g/L)			
Mean (((SD)))	26.3 (6.5)	23.8 (7.3)	0.29
N (%) < 35g/L	6 (100)	94 (95.9)	1
Aspartate aminotransferase (Laboratory normal values: 10–40 IU/L in men and 10–35 UI/L in women)			
Median ((IQR))	39 (29–73)	56 (34–98)	0.08
N (%) > 2N	7 (26.9)	114 (27.2)	0.29
Alanine aminotransferase (Laboratory normal values: 8–45 IU/L in men and 6–35 IU/L in women)			
Median ((IQR))	24 (37–105)	31 (20–50)	0.13
Gamma glutamyl transferase (Laboratory normal values: 9–48 IU/L)			
Median ((IQR))	60 (37–105)	108 (52–220)	0.01
Alkaline phosphatase (Laboratory normal values: 30–125 IU/L)			
Median ((IQR))	110 (76–147)	151 (85–291)	0.06
Lactico Deshydrogenase (LDH) (Laboratory normal values: 190–400 IU/L)			
Median ((IQR))	483 (241–1193)	420 (284–889)	0.72
N (%) > 2N	11(50)	135 (47.7)	0.83
CRP (Laboratory normal values: <6 mg/L)			
Median ((IQR))	43 (26–87)	53 (18–103)	0.57
Ferritin (Laboratory normal values: 24–336 µg/L for men, 11–307 µg/L for women)			
Median ((IQR))	1001 (483–1441)	1224 (554–3110)	0.61
Triglycerides (Laboratory normal values: <1.7 mmol/L)			
Median ((IQR))	1.6 (1.1–2.1)	1.6 (1.2–2.1)	0.80
**Diagnosis and treatment of histoplasmosis**			
Positive serology	0 (0)	6 (11.1)	1
Diagnostic method			
Direct examination	29 (93.5)	165 (51.9)	<0.001
Fungal culture	21 (67.7)	270 (84.9)	0.02
Pathology	26 (83.9)	123 (38.7)	<0.001
Initial treatment			
Deoxycholate amphotericin B	6 (25.8)	39 (43.7)	0.26
Liposomal amphotericin B	2 (19.4)	100 (31.4)	0.003
Itraconazole	21 (67.8)	169 (53.2)	0.11
IV Fluconazole	1 (3.2)	2 (0.6)	0.24
None	1 (3.2)	8 (2.5)	0.57
**Death at 1 month after antifungal initiation**	8/31 (25)	42/318 (13.2)	0.056
